# Structure transformation from Sierpiński triangles to chains assisted by gas molecules

**DOI:** 10.1093/nsr/nwad088

**Published:** 2023-03-30

**Authors:** Chao Li, Zhen Xu, Yajie Zhang, Jie Li, Na Xue, Ruoning Li, Mingjun Zhong, Tianhao Wu, Yifan Wang, Na Li, Ziyong Shen, Shimin Hou, Richard Berndt, Yongfeng Wang, Song Gao

**Affiliations:** Center for Carbon-based Electronics and Key Laboratory for the Physics and Chemistry of Nanodevices, School of Electronics, Peking University, Beijing 100871, China; Institut für Experimentelle und Angewandte Physik, Christian-Albrechts-Universität zu Kiel, Kiel 24098, Germany; Center for Carbon-based Electronics and Key Laboratory for the Physics and Chemistry of Nanodevices, School of Electronics, Peking University, Beijing 100871, China; Center for Carbon-based Electronics and Key Laboratory for the Physics and Chemistry of Nanodevices, School of Electronics, Peking University, Beijing 100871, China; Center for Carbon-based Electronics and Key Laboratory for the Physics and Chemistry of Nanodevices, School of Electronics, Peking University, Beijing 100871, China; Central Laboratory, Tianjin Key Laboratory of Epigenetics for Organ Development in Preterm Infants, the Fifth Central Hospital of Tianjin, Tianjin 300450, China; Center for Carbon-based Electronics and Key Laboratory for the Physics and Chemistry of Nanodevices, School of Electronics, Peking University, Beijing 100871, China; Center for Carbon-based Electronics and Key Laboratory for the Physics and Chemistry of Nanodevices, School of Electronics, Peking University, Beijing 100871, China; Center for Carbon-based Electronics and Key Laboratory for the Physics and Chemistry of Nanodevices, School of Electronics, Peking University, Beijing 100871, China; Center for Carbon-based Electronics and Key Laboratory for the Physics and Chemistry of Nanodevices, School of Electronics, Peking University, Beijing 100871, China; Center for Carbon-based Electronics and Key Laboratory for the Physics and Chemistry of Nanodevices, School of Electronics, Peking University, Beijing 100871, China; Center for Carbon-based Electronics and Key Laboratory for the Physics and Chemistry of Nanodevices, School of Electronics, Peking University, Beijing 100871, China; Center for Carbon-based Electronics and Key Laboratory for the Physics and Chemistry of Nanodevices, School of Electronics, Peking University, Beijing 100871, China; Institut für Experimentelle und Angewandte Physik, Christian-Albrechts-Universität zu Kiel, Kiel 24098, Germany; Center for Carbon-based Electronics and Key Laboratory for the Physics and Chemistry of Nanodevices, School of Electronics, Peking University, Beijing 100871, China; Institute of Spin Science and Technology, South China University of Technology, Guangzhou 511442, China

**Keywords:** scanning probe microscopy, fractals, metal–organic motifs, CO, Sierpiński triangles

## Abstract

Reversible transformations between fractals and periodic structures are of fundamental importance for understanding the formation mechanism of fractals. Currently, it is still a challenge to controllably achieve such a transformation. We investigate the effect of CO and CO_2_ molecules on Sierpiński triangles (STs) assembled from Fe atoms and 4,4″-dicyano-1,1′:3′,1″-terphenyl (C3PC) molecules on Au surfaces. Using scanning tunneling microscopy, we discover that the gas molecules induce a transition from STs into 1D chains. Based on density functional theory modeling, we propose that the atomistic mechanism involves the transformation of a stable 3-fold coordination Fe(C3PC)_3_ motif to Fe(C3PC)_4_ with an axially bonded CO molecule. CO_2_ causes the structural transformation through a molecular catassembly process.

## Teaser

Structure transformations from fractals to chains are realized by the coassemby of carbon monoxide and catassembly of carbon dioxide.

## INTRODUCTION

Many molecules assemble into crystalline or disordered glassy structures on surfaces [1–8]. With certain assembly approaches, specific molecules can be made to form ordered yet aperiodic structures, such as fractals and quasicrystals [9–12]. Sierpiński triangles (STs) have been prepared on surfaces [11,13–24] and in solutions [25–27] through halogen-bonding, metal–organic coordination, hydrogen-bonding and covalent-bonding interactions. 2D arrays of STs were prepared at high molecular coverages [21].

A comprehensive understanding of the growth mechanisms of these fascinating structures is essential for their controlled fabrication on surfaces [28]. Studying their structural transitions may be helpful in this respect. However, reversible transitions between crystalline and fractal structures are still challenging. To induce reversible transformations between fractals and crystalline structures, it appears necessary that the interactions between the assembly units exhibit a certain stability and reversibility. These characteristics can be found in coordination bonds [29,30]. Moreover, the interaction modes between the metal atoms and organic molecules in coordination nodes can be altered by small molecules [31–36]. Recently, H_2_O molecules have been used in the structural transformation and chiral separation of hydrogen-bonded assemblies [37,38]. The mechanism can be explained well by the catassembly theory proposed by Tian's group [39]. Catassembly in molecular assembly is a concept that is analogous to catalysis in chemical synthesis, which refers to the increase in the efficiency and selectivity of assembly processes [39]. These indicate that co-adsorbed molecules might be used to initiate the transition from STs to other structures.

In this work, we prepare stable STs on Au(111) and Au(100) using the coordination between Fe atoms and 4,4″-dicyano-1,1′:3′,1″-terphenyl (C3PC) or 1,3-bis(4-pyridyl)-benzene (BPyB) molecules. Gas molecules (CO and CO_2_) are applied to the ST-covered surfaces to modify the assembly structure. We observe a morphology change from STs into 1D chains with scanning tunneling microscopy (STM). The reverse transition from chains into STs is realized by annealing the samples at ∼350 K. Density functional theory (DFT) calculations reveal the mechanism that the structural transformation is induced by co-assembly of CO and catassembly of CO_2_, respectively.

## RESULTS AND DISCUSSION

### Structure transformation from STs to chains

**Figure 1.**
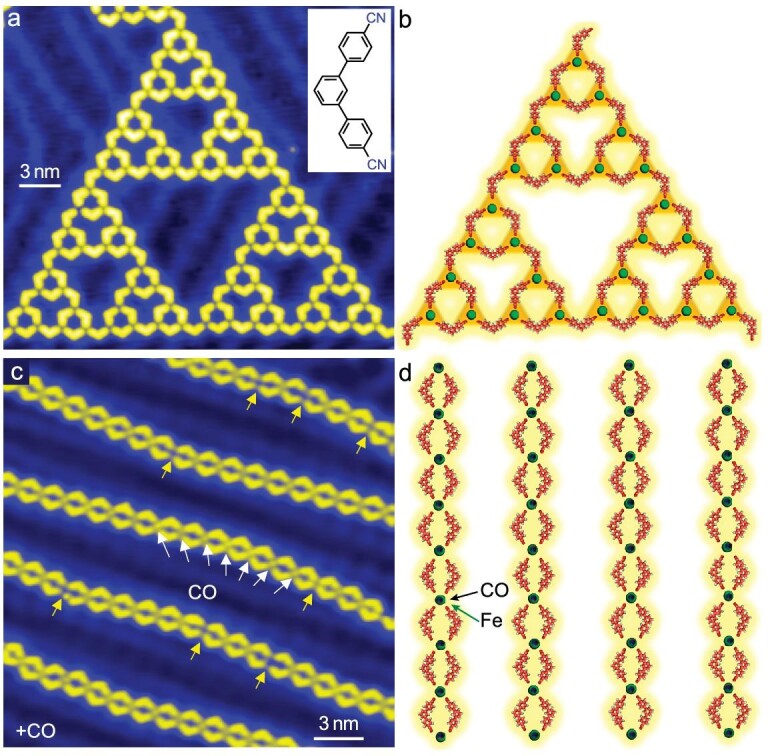
Structure transformation from STs into chains induced by CO molecules. (a) A fourth-order ST formed by Fe atoms and C3PC molecules on Au(111). Inset: chemical structure of C3PC (28 nm × 25 nm). (b) Molecular structures of STs. Green balls represent Fe atoms. (c) Regular long chains obtained after dosing CO on the sample with the pressure of 5.0 × 10^−6^ torr for 120 seconds at room temperature (30 nm × 30 nm). Some CO molecules are marked by white arrows. All Fe atoms without CO molecules are marked by yellow arrows. (d) Molecular structures of long chains. The Fe atoms and CO molecules are marked by green and black arrows, respectively. Imaging parameters: (a) sample voltage *V* = 10 mV, current *I* = 100 pA; (c) *V* = 10 mV, *I* =40 pA.

We prepare STs from C3PC molecules and Fe atoms co-deposited on Au(111) by annealing at ∼350 K for 10 minutes. Figure 1a shows a typical STM image of a fourth-order ST. STs with the order from 0 to 4 are presented in the supporting information (Supplementary Fig. S1). A large-scale image is shown in Supplementary Fig. S2a. As the building block of the ST, three C3PC molecules coordinate via Fe–N bonds with a Fe atom in a 3-fold symmetric fashion (Fig. 1b). The fourth-order ST consists of 81 Fe atoms and 120 C3PC molecules, and covers an area of ∼80 nm^2^.

After dosing CO on the sample with a pressure of 5.0 × 10^−6^ for 120 seconds (600 Langmuir (L)) at ambient temperature, regular long chains packed roughly along < −211 > directions of Au(111) are obtained, as shown in Fig. 1c and Supplementary Fig. S2b. Close inspection of the STM image of the long chains reveals protrusions that are preferentially located at the iron atoms that serve as coordination nodes. In contrast, the protrusions are not observed at the Fe positions in STs. We attribute them to axially bonded CO molecules, marked by white arrows. After applying a voltage pulse at 2.0 V, CO can be desorbed from the Fe atom (Supplementary Fig. S3). No significant amounts of CO are observed on the bare Au(111) surface, which is consistent with the easy desorption of CO from Au(111) at ambient temperature [40]. Figure 1d shows the molecular structure of the regular chain pattern. The basic motif consists of four C3PC molecules bonding to an Fe coordination center that in turn binds a CO molecule axially. A 3-fold Fe–N coordination bond has been proven to be the most stable form after sufficient annealing of the sample [13,14,16,19–21]. The emergence of four Fe–N coordination bonds with an axial Fe–C bond is unexpected.

### CO exposure at different dosages and temperatures

**Figure 2.**
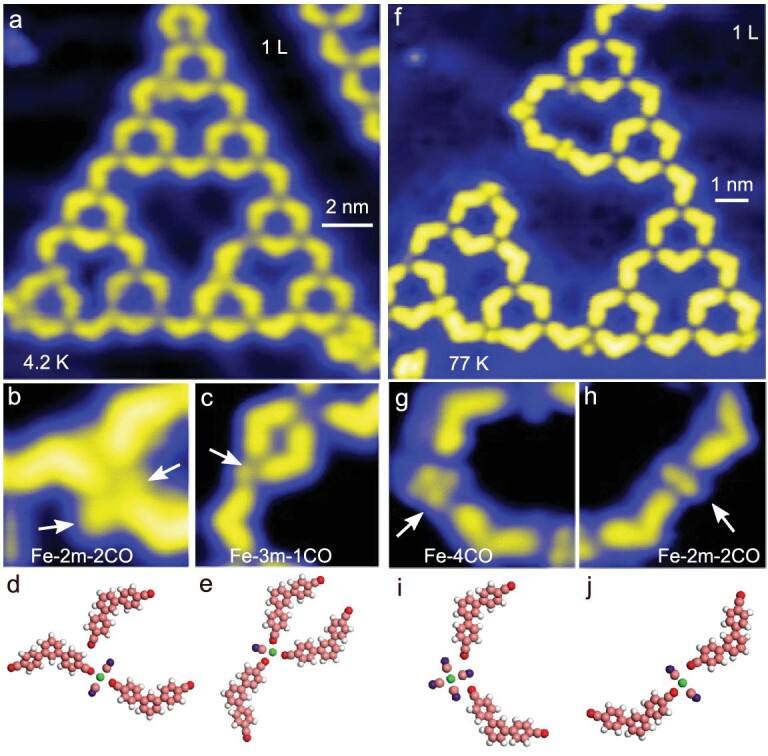
CO exposure at different doses and temperatures. (a) Third-order ST after dosing CO with a pressure of 1.0 × 10^−8^ for 100 seconds (1 L) at ∼30 K. (b–e) Enlarged STM images of two representative Fe(C3PC)_3_ motifs in the presence of CO and corresponding models. CO molecules are marked by white arrows. (f) Third-order ST after dosing CO with a pressure of 1.0 × 10^−8^ for 100 seconds (1 L) at ∼100 K. (g–j) Two representative motifs with CO and corresponding models. Imaging parameters: (a) 13 nm × 13 nm; (b) 3 nm × 3 nm; (c) 3.4 nm × 3.4 nm; (f) 11 nm × 11 nm; (g) 3.1 nm × 3.1 nm; (h) 3.3 nm × 3.3 nm. (a) Sample voltage *V* = 0.1 V, current *I* = 40 pA; (b) and (c) *V* = 5 mV, *I* = 40 pA; (f) *V* = –10 mV, *I* = 20 pA; (g) and (h) *V* = –5 mV, *I* = 20 pA.

To get insight into the mechanism of the CO-induced transformation, further data are acquired at low CO dosages and low temperatures. After exposing STs to CO with the pressure of 1.0 × 10^−8^ for 100 seconds (1 L) at ∼30 K, a series of subtle structural changes of the ST are observed (Fig. 2a and Supplementary Fig. S4a). In particular, previously absent, round features close to the C3PC molecules and the Fe coordination centers are observed (Figs 2b and c, white arrows). In both Fig. 2b and c, CO molecules appear to interact with an Fe atom. In Fig. 2b, two CO molecules are inserted into the motif at an Fe atom. A previously coordinated ligand seems to have been pushed away, as is evident from the widely increased distance of the ligand center from the Fe atom. The distance is 400 pm larger than for other ligands, preventing any direct bonding to the Fe center. The structures proposed in Fig. 2d and e model the adjacent images (Fig. 2b and c).

After dosing CO of the same amount on STs at ∼100 K, a changed surface morphology is experimentally observed (Supplementary Fig. S4b). As shown in Fig. 2f, the outline of a third-order ST is no longer a regular triangle, and some distorted and imperfect patterns have appeared. The magnified image in Fig. 2g shows the disruption of an array by CO molecules. The model in Fig. 2i indicates that the Fe atom is completely surrounded by four CO molecules, and exhibits no bonds to C3PC ligands. Figure 2h (model in Fig. 2j) shows a related case in which the center Fe atom binds two CO molecules and two C3PC ligands. A voltage pulse at 2 V leads to the desorption and diffusion of CO molecules (Supplementary Fig. S5).

### Mechanism of transformation from STs to chains induced by CO

**Figure 3.**
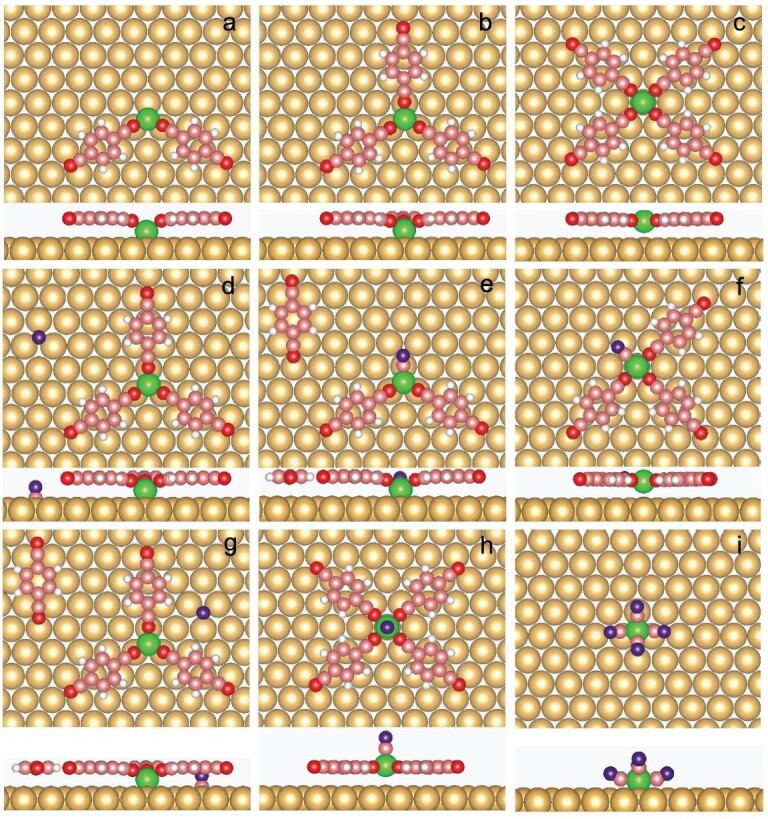
Top and side views of optimized structures from DFT calculations for motifs consisting of an Fe center (light brown), CPC ligands (blue and brown) that are used instead of C3PC to simplify the calculations and CO (brown and red) on a Au(111) substrate (yellow). (a) Fe(CPC)_2_, (b) Fe(CPC)_3_, (c) Fe(CPC)_4_, (d) Fe(CPC)_3_ and one physisorbed CO, (e) FeCO(CPC)_2_ and one physisorbed CPC, (f) FeCO(CPC)_3_, (g) Fe(CPC)_3_ and one CPC and one CO, (h) FeCO(CPC)_4_, (i) Fe(CO)_4_.

Spin-polarized DFT calculations are performed to investigate the mechanism of the transformation from STs to chains. A NC–Ph1–CN ligand (CPC) is used to represent C3PC for simplicity (Fig. 3a). The calculated binding energies of Fe(CPC)_2_, Fe(CPC)_3_ and Fe(CPC)_4_ are –1.84, –2.57 and –2.32 eV, respectively. As shown in Fig. 3a–c, the favorable adsorption sites for Fe atoms are fcc, fcc and top sites, separately. The calculated result indicates that 3-fold coordinated nodes are energy-favorable and this explains why STs are experimentally observed after annealing processes. The 5-fold coordinated Fe(CPC)_5_ is energetically unstable and it changes into Fe(CPC)_4_ and an isolated CPC after optimization.

Compared with Fe(CPC)_3_ along with one physisorbed CO (Fig. 3d), a structure with CO replacing a CPC (Fig. 3e) gains an energy of 0.58 eV. Once 4-fold coordination is formed, FeCO(CPC)_3_ (Fig. 3f) is more favorable by 0.15 eV. In other words, CO prefers to attach to Fe centers. When one Fe(CPC)_3_ attaches one CPC and one CO (Fig. 3g and h), the final product FeCO(CPC)_4_ gets more stable with an energy of 0.41 eV. The calculated binding energy of FeCO(C3PC)_4_ is –2.67 eV, which is larger than that of Fe(C3PC)_3_ (–2.57 eV). The interaction energy between Fe(CPC)_4_ and CO is 0.71 eV. It means that the chain structures most likely benefit from CO binding axially to Fe. After dosing CO molecules, some Fe atoms are detached from STs and fully coordinated by CO molecules. Fe(CO)_4_ (Fig. 3i) is found in experiments and its calculated binding energy is –2.8 eV.

Based on STM and DFT data, we suggest that CO molecules play pivotal roles in the structural transformation from STs to chains (Supplementary Fig. S6). At first, CO has a strong tendency to bind to Fe and changes 3-fold Fe(C3PC)_3_ to 4-fold Fe(CO)_x_ (C3PC)_4__–__x_. Second, CO molecules can replace C3PC from coordinated structures. These freely diffused C3PC molecules interact with Fe(CO)_x_ (C3PC)_4__–__x_ and form the final product FeCO(C3PC)_4_, which is the building block of the chains on Au(111). Finally, CO molecules stabilize the Fe atoms released from STs by forming Fe_x_(CO)_y_ clusters, such as Fe(CO)_4_. The fact that different highly ordered patterns may be achieved in the present case may be related to the openness of the fractal structure (Hausdorff dimension 1.59), which leaves ample space for rearrangements of the ligands.

In the next step, we will discuss the mechanism of transformation from STs to chains from the perspective of dynamics. The transition process can be simplified by using Equation (1):


(1) \begin{eqnarray*} {\rm{Fe}}\left( {{\rm{C}}3{\rm{PC}}} \right)_3{\rm{\ }} + {\rm{\ CO\ }} \to {\rm{\ FeCO}}\left( {{\rm{C}}3{\rm{PC}}} \right)_4\\ {\rm{\ }} + {\rm{\ Fe_x}}\left( {{\rm{CO}}} \right)_{\rm{y}} \end{eqnarray*}

In STs, Fe and C3PC ligands form 3-fold coordination bonds. In chains, an Fe atom coordinates with four C3PC ligands laterally and binds to one CO molecule axially. Partial Fe atoms are fully coordinated by CO and they form Fe_x_(CO)_y_ clusters, which appear as bright protrusions in STM images (Supplementary Figs S2b and S3). According to Le Chatelier’s principle, more chain structures can be obtained when increasing the amount of CO. This is consistent with our experimental results.

In summary, CO facilitates the transition from 3-fold coordination to the CO–Fe(C3PC)_4_ pattern in chains energetically. By increasing the dosage of CO, chains are favored on Au(111) dynamically. Further Monte Carlo modeling is suggested to get a better understanding of the co-assembly process [41].

### Structure transformation assisted by CO_2_ on different substrates

**Figure 4.**
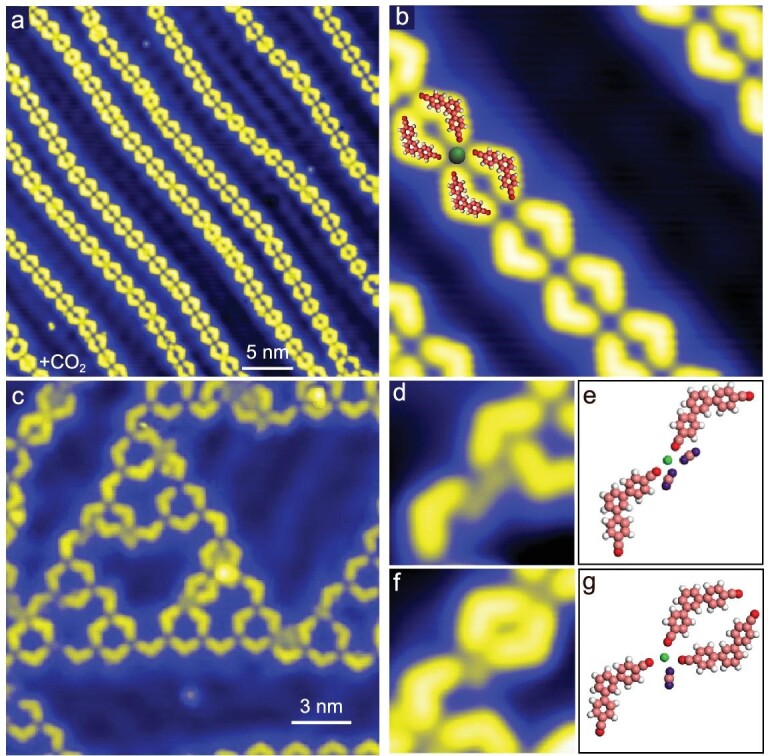
CO_2_-induced structure transition on Au(111). (a) A surface covered with the four-coordination regular long chains after dosing CO_2_ of 650 L on STs/Au(111) (36 nm × 36 nm). (b) Enlarged high-resolution STM image of the long chains (8.1 nm × 8.1 nm). (c) CO_2_ exposure with 60 L on Fe–C3PC–STs/Au(111) at ∼30 K. A distorted third-order ST (18 nm × 18 nm). (d) and (e) Two CO_2_ molecules attach to a Fe atom, forcing the original C3PC to interact with its upper C3PC (3.4 nm × 3.4 nm), and corresponding adsorption model. (f) and (g) Three C3PC and a CO_2_ are attached to an Fe atom (3.2 nm × 3.2 nm) and corresponding adsorption model. Imaging parameters: *I* =0.04 nA; (a) and (b) *V* = 10 mV, (c) *V* = 0.1 V, (d) *V* = 5 mV, (f) *V* = 10 mV.

To study the effect of gas molecules, we repeat experiments with CO_2_. A series of large-scale images are shown in the supporting information (Supplementary Fig. S7) to show the sequential structural transition from STs to chains induced by CO_2_ molecules. Figure 4a shows a large-scale STM image of regular long chains recorded after dosing CO_2_ with a pressure of 5.0 × 10^−6^ for 130 seconds (650 L) at ambient temperature on a ST sample. Long regular chains are formed roughly along <−211> directions. In other words, the chains are parallel to the ridges of the herringbone reconstruction of Au(111). The enlarged STM image in Fig. 4b reveals that each Fe atom is coordinated with four C3PC molecules. Compared with chains induced by CO, much fewer protrusions are found at Fe atoms. By analogy with catalysis in chemical reactions, catassembly is suggested in the molecular assembly process. Similar to a catalyst, a catassembler increases the efficiency or selectivity of an assembly process, but does not exist in the final assembly structures [39]. Here, CO_2_ molecules perform like catassemblers, which induce the transformation from STs to chains but are not present in the final structure.

We further extend CO_2_ exposure to STs on Au(100) to understand the substrate effect in the structural transformation; C3PC molecules coordinate with Fe atoms and induce 1D double chains of ST-2 after deposition and 5 minutes of annealing at 350 K (Supplementary Fig. S8a and S8b) [19]. The structure is also stabilized through 3-fold Fe–N bonds. The reconstructed rows of Au(100) play an important role in the structural formations. Similar to the effect of CO_2_ on STs on Au(111), a dose of 650 L CO_2_ at room temperature leads to a collapse of 1D double chains of ST-2 and the formation of a new type of long chain adsorbed along the reconstructed rows (Supplementary Fig. S8c and S8d). CO_2_ molecules induce the transition of coordination nodes from Fe(C3PC)_3_ to Fe(C3PC)_4_. The newly formed chains consist of Fe(C3PC)_4_ units, which is similar to that on Au(111).

To get an insight into the transformation from STs to chains, CO_2_ molecules are dosed at small amounts and low temperatures on STs. At ∼30 K, a clear change of the 3-fold Fe(C3PC)_3_ units is observed only until the CO_2_ dosage reaches 60 L, which is significantly larger than that of CO (1 L). As shown in Fig. 4c, the outline of a third-order ST is no longer a regular triangle and some distorted motifs have appeared in the ST. An enlarged STM image in Fig. 4d shows the disruption of the motif by two attached CO_2_ molecules. A C3PC is expelled from the Fe center and interacts with the upper C3PC through hydrogen bonds; the corresponding adsorption model is shown in Fig. 4e. A number of similarly deformed motifs lead to a distortion of the entire ST. The enlarged STM image in Fig. 4f and its corresponding adsorption model in Fig. 4g present another type of intermediate structure in which an Fe atom coordinates with three ligands and a CO_2_ molecule. The voltage pulse at 2 V can induce molecular diffusion, which proves that the bright spots are CO_2_ molecules instead of Fe atoms (Supplementary Fig. S9).

### Mechanism of transformation from STs to chains induced by CO_2_

The experimental results indicate that CO_2_ leads to the structural transformation from STs to chains through a catassembly process (Supplementary Fig. S10). It is simplified by using Equation (2):


(2) \begin{eqnarray*} {\rm{Fe}}\left( {{\rm{C}}3{\rm{PC}}} \right)_3{\rm{\ }} + {\rm{\ CO}}_2{\rm{\ }} \to {\rm{\ Fe}}\left( {{\rm{C}}3{\rm{PC}}} \right)_4\\ {\rm{\ }} + {\rm{\ Fe_x}}\left( {{\rm{CO}}2} \right)_{\rm{y}} \end{eqnarray*}

**Figure 5.**
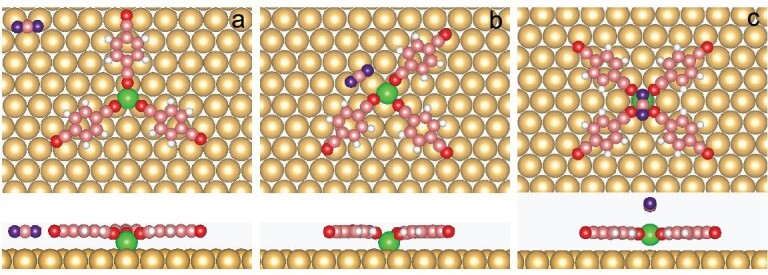
DFT-calculated interactions between CO_2_ and Fe(CPC)_3_ and Fe(CPC)_4_. (a) and (b) The structure of Fe(CPC)_3_ with a physisorbed CO_2_ is more stable than Fe(CPC)_3_CO_2_ with an energy of 0.06 eV. (c) The binding energy between axially bonded CO_2_ and Fe is 0.16 eV in Fe(CPC)_4_CO_2_.

The interactions between CO_2_ and Fe(CPC)_3_ and Fe(C3PC)_4_ are calculated to understand the process of the structural transition. Similar to the effect of CO, the structural transition here is also mediated by CO_2_-induced changes in the coordination shell of Fe centers. Figure 5b presents the optimized structure of Fe(CPC)_3_CO_2_, which is less stable than the structure of Fe(CPC)_3_ and a physisorbed CO_2_ (Fig. 5a) with an energy of 0.06 eV. In Fe(CPC)_4_CO_2_ (Fig. 5c), the binding energy between axially bonded CO_2_ and Fe is only 0.16 eV, which is much lower than the binding energy of 0.71 eV between axially bonded CO and Fe (Fig. 3h). CO_2_ molecules desorb easily from Fe atoms. This explains why CO_2_ does not exist in the chains (Fig. 4b).

Based on the calculated results, we suggest the following mechanism of the structural transformation from STs into chains induced by CO_2_. The catassembly process is energetically unfavorable. So, the energy change is not the driving force for the structure transition. According to Le Chatelier’s principle, CO_2_ can interact with 3-fold Fe(C3PC)_3_ coordination nodes, which leads to the formation of 4-fold Fe(C3PC)_x_(CO_2_)_4__–__x_ nodes at a large dosage of CO_2_. When CO_2_ molecules are replaced by diffused C3PC molecules, chains comprising Fe(C3PC)_4_ are finally formed. A large dosage of CO_2_ plays the key role in the structure transition from STs into chains.

To testify the effect of the interaction between CO_2_ and coordination nodes on the structural transformation, C3PC molecules are replaced by 4,4′-(1,3-phenylene)dipyridine (BPyB) molecules Supplementary Fig. S11a presents a fourth-order Fe–BPyB–ST stabilized by coordinated Fe–N bonds. Protrusions are observed close to molecules after dosing CO_2_ of 60 L at ∼30 K (Supplementary Fig. S11b). Different to Fe(C3PC)_3_, the Fe(BPyB)_3_ nodes keep intact after dosing CO_2_ of the same amount. This implies that it is difficult to change BPyB–STs into chains by using CO_2_ molecules. As expected, only a small number of Fe–BPyB–STs are transformed into 4-fold coordinated chains by the same dosage (650 L) of CO_2_ compared with Fe–C3PC–STs (Supplementary Fig. S11c). An enlarged STM image of a short Fe(BPyB)_4_ chain is shown in Supplementary Fig. S11d, which is highlighted by the white dashed rectangle. For Fe(C3PC)_3_ and Fe(BPyB)_3_, they are both stabilized by Fe–N coordinated bonds. Therefore, their dissimilar response to CO_2_ molecules should not be due to the energy difference. Their optimized structures are displayed in Supplementary Fig. S11e and f. The nearest distances between two hydrogen atoms of neighbor molecules are 0.18 nm for Fe(BPyB)_3_ and 0.62 nm for Fe(C3PC)_3_, respectively. Considering the size of CO_2_, we suggest that the steric restriction of BPyB makes it difficult for CO_2_ to interact with Fe, which reduces the probability of structural transformation from STs into chains.

### Spin states of Fe atoms in STs and chains

**Figure 6.**
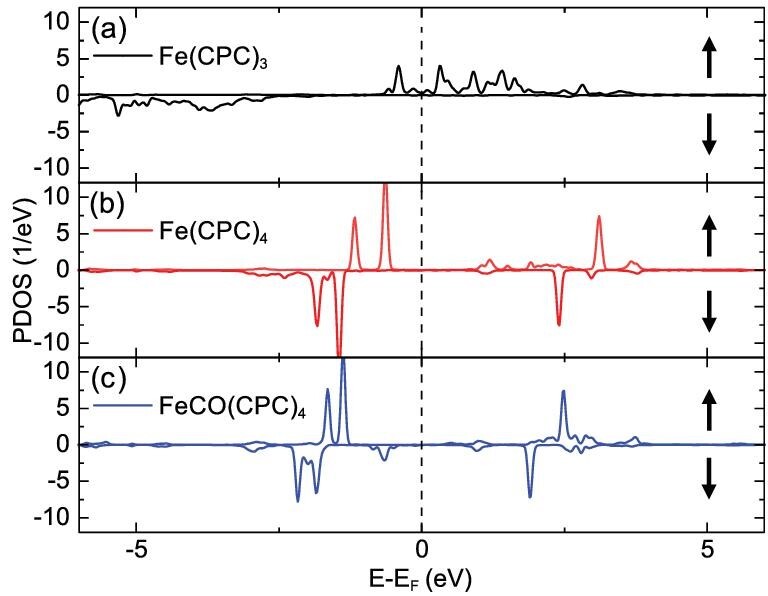
Spin-polarized density of states projected onto the Fe d-orbitals for the coordination motifs (a) Fe(CPC)_3_, (b) Fe(CPC)_4_ and (c) FeCO(CPC)_4_. Their calculated magnetic moments are 3.6, 1.2 and 0.9 *μ*_B_, respectively.

It is attractive to investigate the magnetic properties of Fe atoms in STs and chains. Spin states of Fe atoms in Fe(CPC)_3_, Fe(CPC)_4_ and FeCO(CPC)_4_ are calculated, as shown in Fig. 6. Their magnetic moments are 3.6, 1.2 and 0.9 *μ*_B_, respectively. For Fe(CPC)_3_, the electronic system of Fe may be described as the approximate d^6^ configuration. There are around five electrons in the majority spin state (↓) and only one electron in the minority spin state (↑), leading to a total spin *S* = 2. For both Fe(CPC)_4_ and FeCO(CPC)_4_, there are four electrons in the majority spin state and three electrons in the minority spin state. As a consequence, their spin configurations approximately correspond to *S* = 1/2. This spin configuration is similar to that of FePc adsorbed on Ag(100) [42]. Unfortunately, no spin-related Kondo or inelastic tunneling signals are observed when performing dI/dV measurements using STM, which might be due to a small contribution to currents from spin states. Other experimental methods such as X-ray Magnetic Circular Dichroism (XMCD) are suggested to detect spin states of Fe atoms in STs and chains.

## CONCLUSION

In summary, structural transformations from STs to chains assisted by CO and CO_2_ molecules are investigated by low-temperature STM and DFT calculations. C3PC molecules and Fe atoms form STs on Au(111) through 3-fold coordination interactions. After dosing with CO molecules, the stable coordination Fe(C3PC)_3_ motifs change into Fe(C3PC)_4_ with an axially bonded CO molecule, which transforms STs into 1D chains. The chains are reverted to STs by annealing of samples. The structure transition can also be realized by CO_2_ molecules and on the Au(100) substrate. Different to the co-assembly of CO with Fe(C3PC)_4_, CO_2_ molecules induce the structural transformation through a molecular catassembly process. The co-assembly and catassembly methods might be used to tune other metal–organic structures on surfaces.

## METHODS

### STM characterization and sample preparation

The measurements are performed using a scanning tunneling microscope (UNISOKU, USM-1500) with a base pressure of 1 × 10^−10^ Torr. All STM images are acquired with Pt/Ir tips at a temperature of 4.3 K. Au(111) and Au(100) surfaces are prepared by several cycles of Ar ion sputtering and annealing. Fe atoms, C3PC molecules and BPyB molecules are sublimated onto the substrates at room temperature from different Ta boats. The STM images are processed using the software WSxM [43].

### DFT calculations

Spin-polarized DFT calculations are performed using the Vienna Ab-initio Simulation Package (VASP) [44,45]. The ion–electron interaction is described by the projector augmented wave method [46]. The exchange-correlation potential is described by using the generalized gradient approximation [47]. Calculations are carried out by using PBE + TSvdw functional where the van der Waals dispersion correction is added by the Tkatchenko–Scheffler method [48]. A correlation correction U – J = 3 eV is used to treat d-orbitals of Fe atoms, where U and J correspond to the Coulomb interaction and the exchange coupling, using the scheme by Dudarev *et al.* [49]. The kinetic energy cut-off is set to 500 eV. The first Brillouin zone is sampled with a Γ-centered k-mesh. During the optimization, except for the Au atoms in the two bottom layers of the slabs that are fixed, all other atoms are fully relaxed until the force on each of them is <0.02 eV/Å. The binding energy of the ligands with Fe in the presence of Au(111) substrate is calculated by using the formula Δ*E* = *E*_Fe(CPC)n/sub_ – *E*_Fe/sub_ – n*E*_CPC/sub_ + n*E*_sub_, where *E*_Fe(CPC)n/sub_, *E*_Fe/sub_, *E*_CPC/sub_ and *E*_sub_ refer to the energies of the Fe(CPC)_n_ motif on the substrate, one Fe adatom with the most stable fcc adsorption site on the substrate, one CPC molecule on the substrate and the Au(111) substrate, respectively. The total binding energy of FeCO(CPC)_4_ on Au(111) is calculated by using the formula Δ*E* = *E*_FeCO(CPC)4/sub_ – *E*_Fe/sub_ – 4*E*_CPC/sub_ – *E*_CO/sub_ + 5*E*_sub_. The binding energy between CO/CO_2_ and Fe(CPC)_4_ on Au(111) is calculated by using the formula Δ*E* = *E*_system_ – *E*_Fe(CPC)4/sub_ – *E*_CO/CO2_, where *E*_system_ refers to the total energy of the FeCO(CPC)_4_ or FeCO_2_(CPC)_4_ adsorbed on Au(111).

## Supplementary Material

nwad088_Supplemental_File
