# Supplementary material for: Structure transformation from Sierpiński triangles to chains assisted by gas molecules
Source: Natl Sci Rev. 2023 Mar 30;10(7):nwad088. doi: 10.1093/nsr/nwad088 (PMC10411674; doi:10.1093/nsr/nwad088)
Supplement: nwad088_Supplemental_File [file nwad088_supplemental_file.docx]

**SUPPORTING INFORMATION FOR**

Structure transformation from Sierpiński triangles to chains assisted by gas molecules

Chao Li, ^‡ [a][b]^ Zhen Xu, ^‡ [a]^ Yajie Zhang, ^‡* [a]^ Jie Li,^[a]^ Na Xue,^[c]^ Ruoning Li, ^[a]^ Mingjun Zhong,^[a]^ Tianhao Wu,^[a]^ Yifan Wang,^[a]^ Na Li,^[a]^ Ziyong Shen,^[a]^ Shimin Hou, ^* [a]^ Richard Berndt, ^* [b]^ Yongfeng Wang,^*[a]^ Song Gao^[d]^

^[a]^ Center for Carbon-based Electronics and Key Laboratory for the Physics and Chemistry of Nanodevices, School of Electronics, Peking University, Beijing 100871, China

^[b]^ Institut für Experimentelle und Angewandte Physik, Christian-Albrechts-Universität zu Kiel, Leibnizstraße 19, 24098 Kiel, Germany

^[c]^ Central Laboratory, Tianjin Key Laboratory of Epigenetics for Organ Development in Preterm Infants, the Fifth Central Hospital of Tianjin, Tianjin 300450, China

^[d]^ Institute of Spin Science and Technology, South China University of Technology, Guangzhou 511442, China

*Correspondence to: yongfengwang@pku.edu.cn, berndt@physik.uni-kiel.de, yjzhang11@pku.edu.cn and smhou@pku.edu.cn.

†These authors contributed equally to this work.


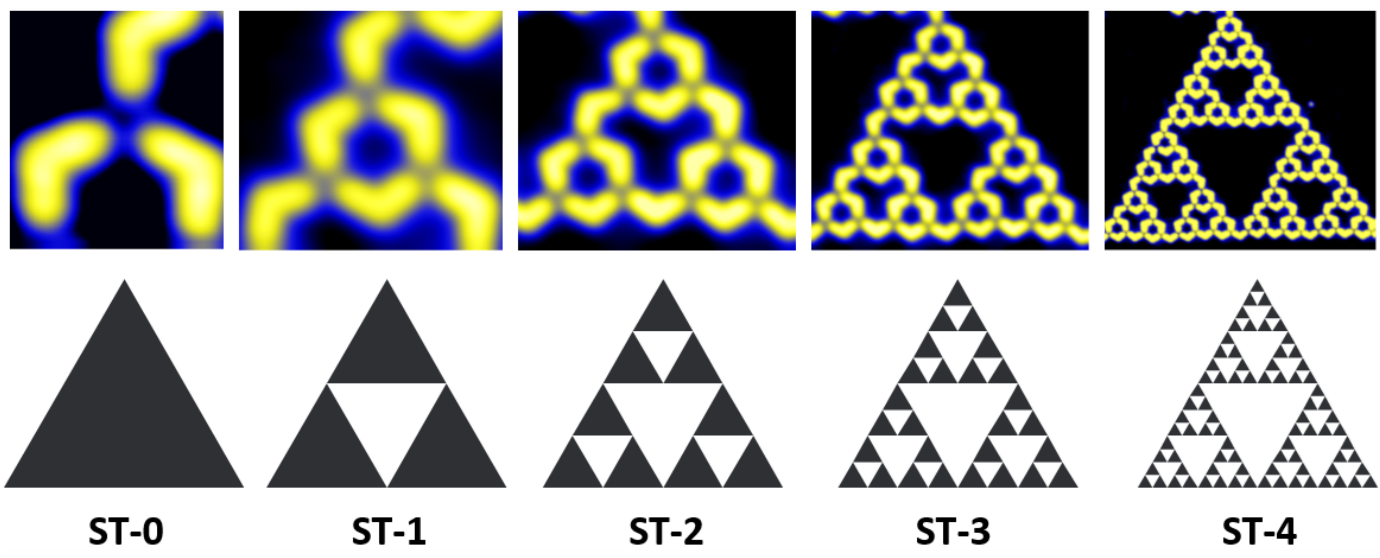


**Figure S1.** STM images of Fe-C3PC-STs-n (n = 0, 1, 2, 3 or 4) and their corresponding models. An ST-n is comprised of three STs-(n-1).

**
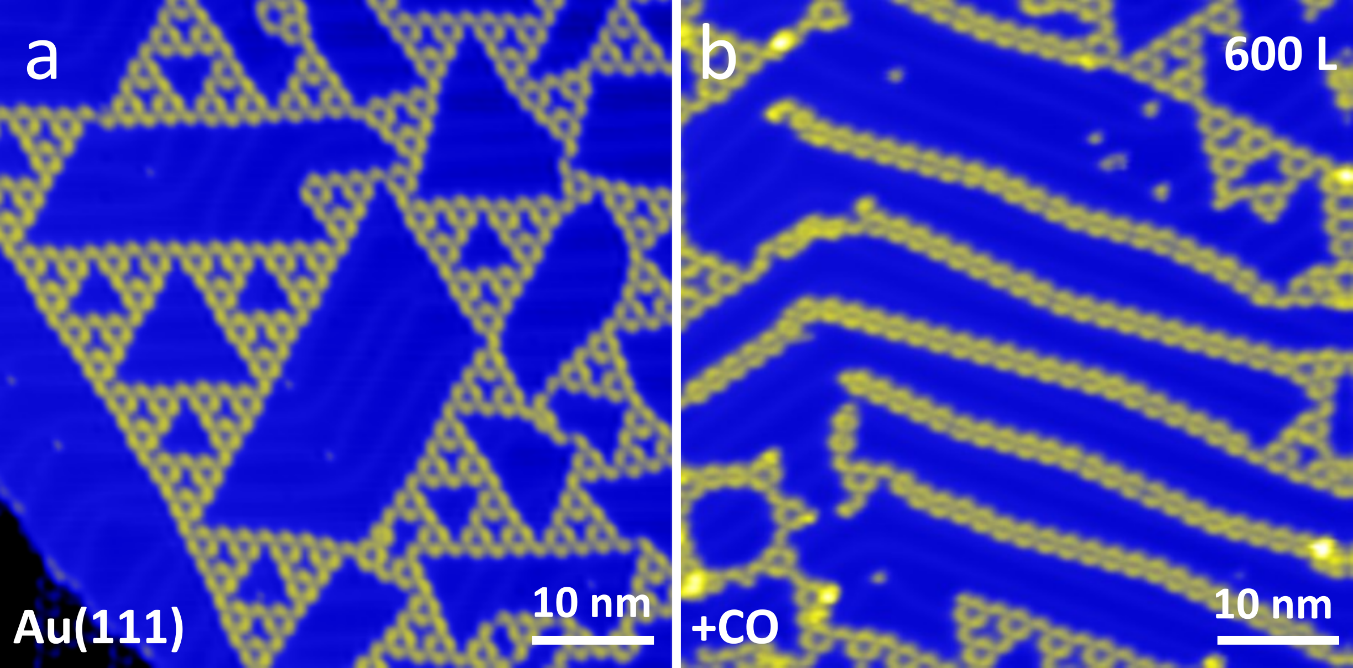
**

**Figure S2. (a)** Large-scale STM image of STs formed by C3PC molecules and Fe atoms on Au(111) (55 nm × 55 nm). (**b**) Large-scale STM image of chains obtained after dosing CO of 600 L on the sample at room temperature (55 nm × 55 nm). Imaging conditions: (a) *V*_bias_ = 1 V, *I* = 0.02 nA; (b) *V*_bias_ = 1 V, *I* = 0.04 nA.


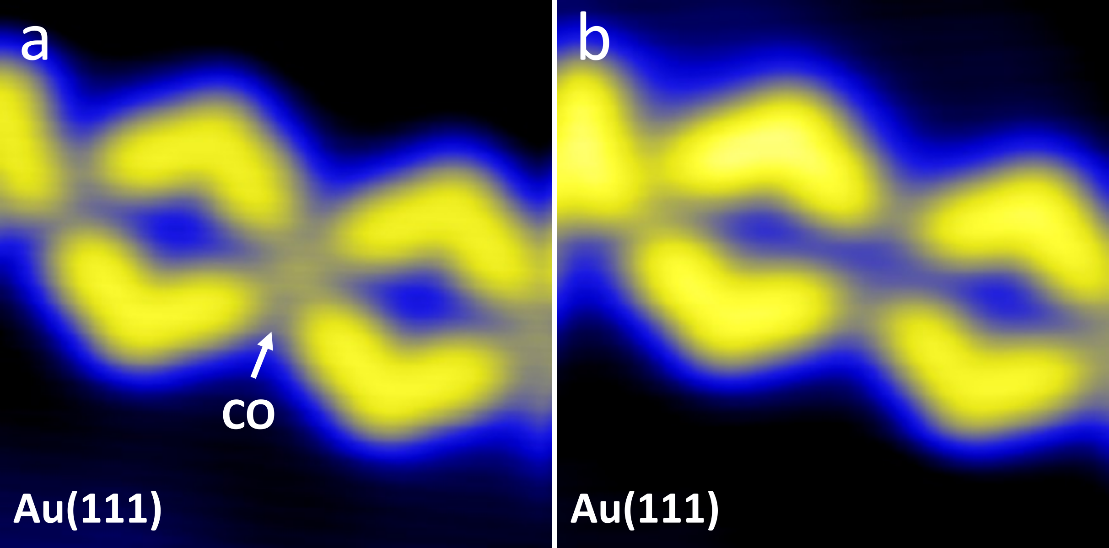


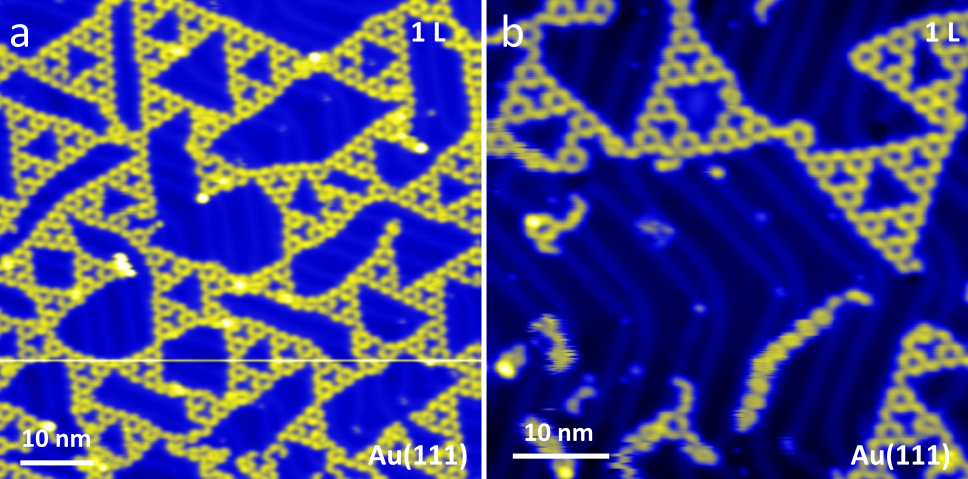
**Figure S3.** A pristine CO molecule adsorbed on top of an C3PC-coordinated Fe atom marked by white arrow (**a**) is removed by a voltage pulse at 2 V (**b**). Imaging parameters for both images: 4 nm × 4 nm; *V*_bias_ = 0.5 V, *I* = 0.04 nA.

**Figure S4. (a)** Large-scale STM image of a Fe-C3PC-ST after dosing CO with the pressure of 1.0 × 10^−8^ for 100 seconds (1 L) at approximately 30 K on Au(111) (70 nm × 70 nm). (**b**) Large-scale STM image of a ST after dosing CO with the pressure of 1.0 × 10^−8^ for 100 seconds (1 L) at around 100 K on Au(111) (50 nm × 50 nm). Imaging parameters: (a) *V*_bias_ = 0.5 V, *I* = 0.04 nA; (b) *V*_bias_ = -0.5 V, *I* = 0.03 nA.


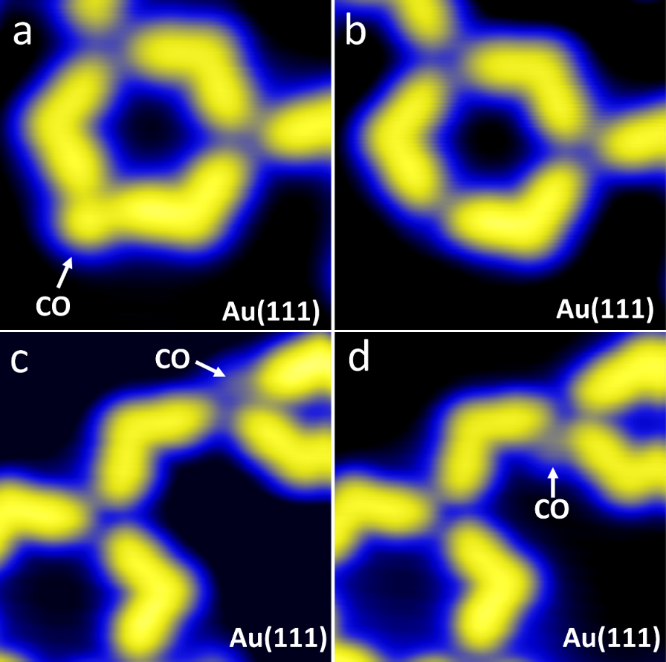


**Figure S5. (a-b)** A pristine CO molecule adsorbed at an Fe atom marked by a white arrow is removed by a voltage pulse at 2 V. (**c-d**) A pristine CO molecule adsorbed at an Fe atom marked by a white arrow moves to another position after applying a voltage pulse at 2 V. Imaging parameters for all images: 3.7 nm × 3.7 nm; *V*_bias_ = 10 mV, *I* = 0.04 nA.


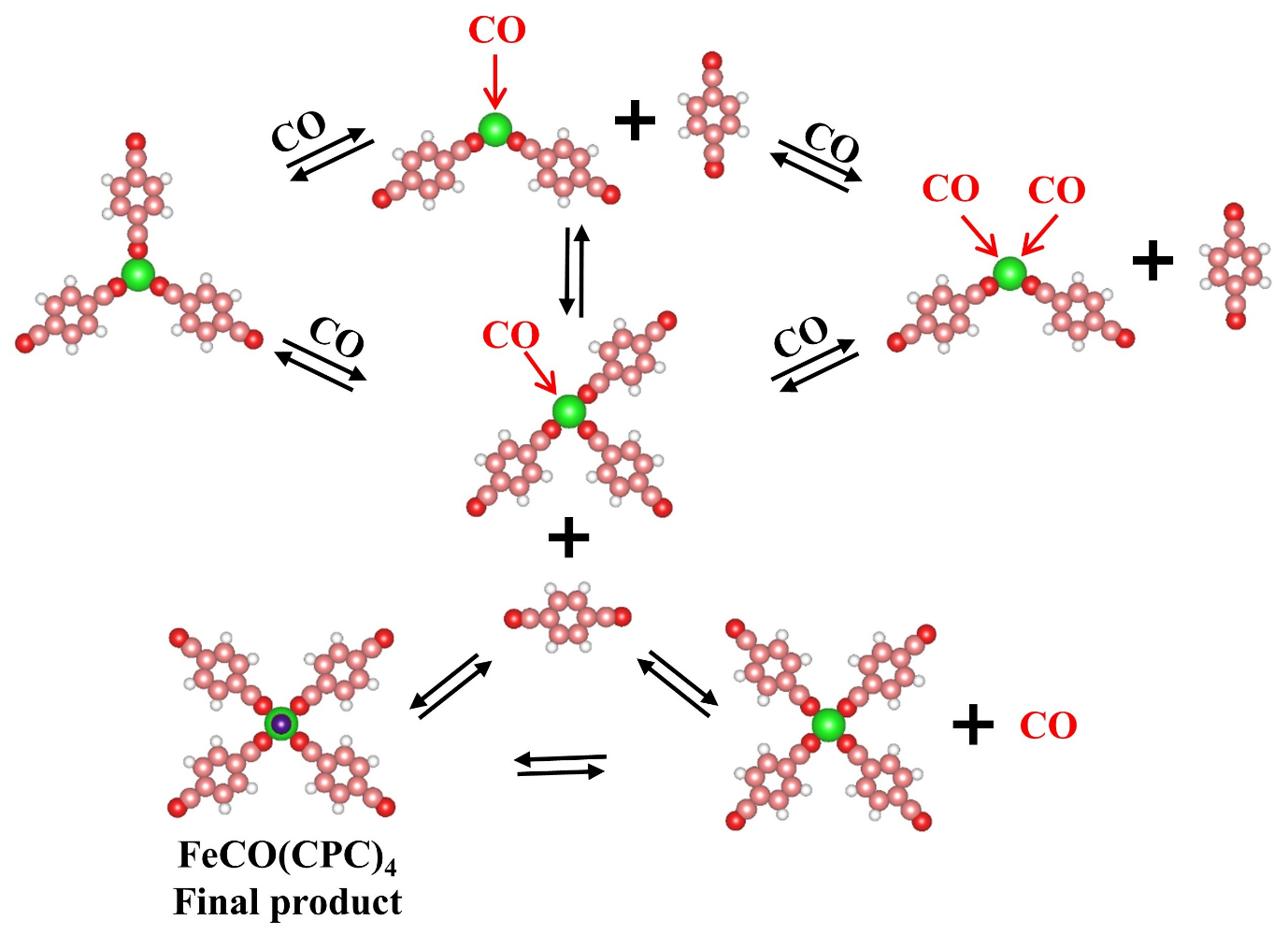


**Figure S6.** Scheme of structure transition from Fe(CPC)_3_ to FeCO(CPC)_4_, which is used to understand transformation from STs to chains via the CO assembly process. According to Le Chatelier′s Principle, the final product is favorable when increasing the amount of CO.


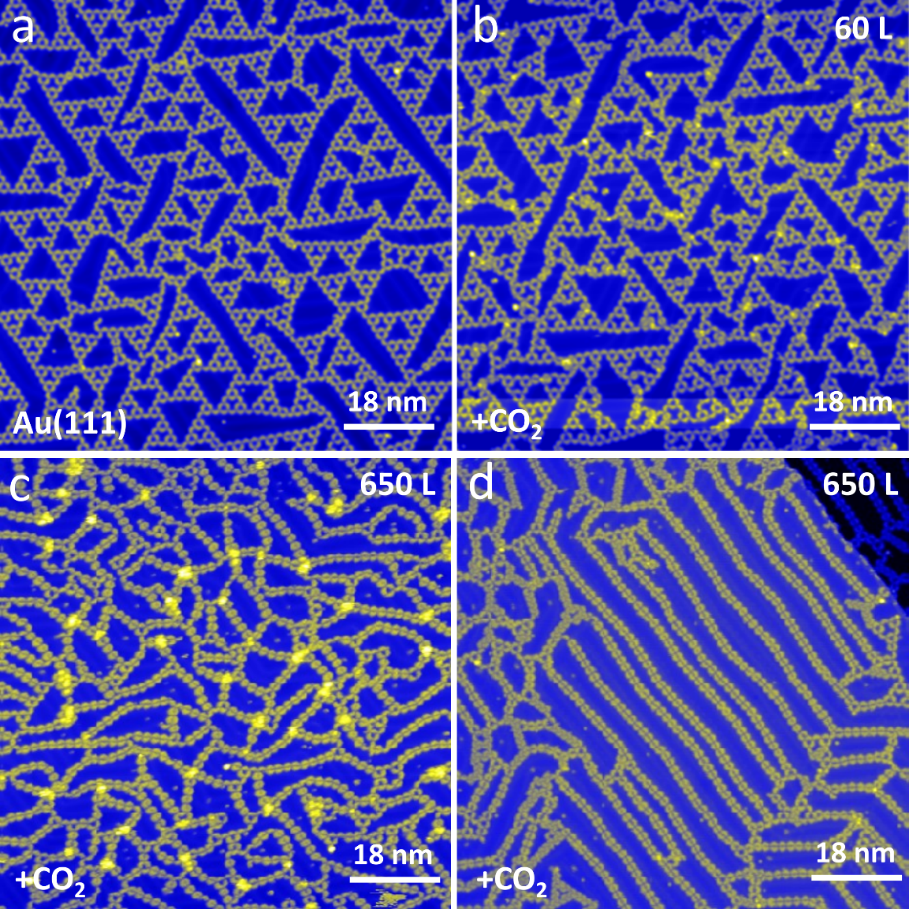
**Figure S7. (a)** Large-scale STM image of a Fe-C3PC-ST formed by C3PC molecules and Fe atoms on Au(111). (**b**) Large-scale STM image of CO_2_ exposure with 60 L on the ST at around 30 K. (**c**) Large-scale STM image of a surface covered with the disorder distributed 4-coordination chains after dosing CO_2_ of 650 L on STs at around 100 K. (**d**) Large-scale STM image of a surface covered with the 4-coordination regular long chains after dosing CO_2_ of 650 L on the ST at ambient temperature. Imaging parameters for all images: 90 nm × 90 nm, *V*_bias_ = 1 V, *I* = 0.04 nA.


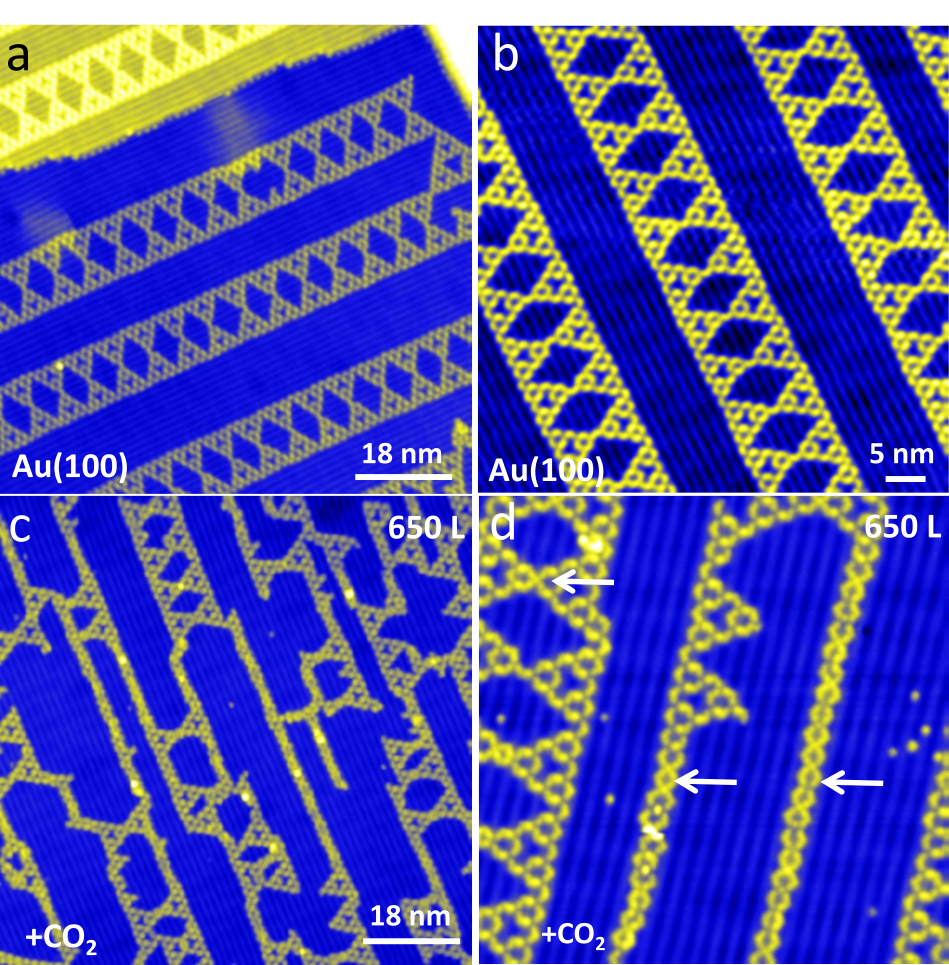
**Figure S8. (a)** Large-scale STM image of double chains of ST-2 formed by C3PC molecules and Fe atoms on Au(100) (90 nm × 90 nm). (**b**) Enlarged STM image of double chains of the ST-2 on Au(100) (58 nm × 58 nm). (**c**) Large-scale STM image of four-fold coordination chains formed after dosing CO_2_ of 650 L on the ST (90 nm × 90 nm). (**d**) Enlarged STM image of four-fold coordination chains as highlighted by white arrows (36 nm × 36 nm). Imaging parameters: (a) *V*_bias_ = 1 V, *I* = 0.05 nA; (b) *V*_bias_ = 0.1 V, *I* = 0.05 nA; (c) *V*_bias_ = 0.1 V, *I* = 0.04 nA; (d) *V*_bias_ = 0.1 V, *I* = 0.05 nA.


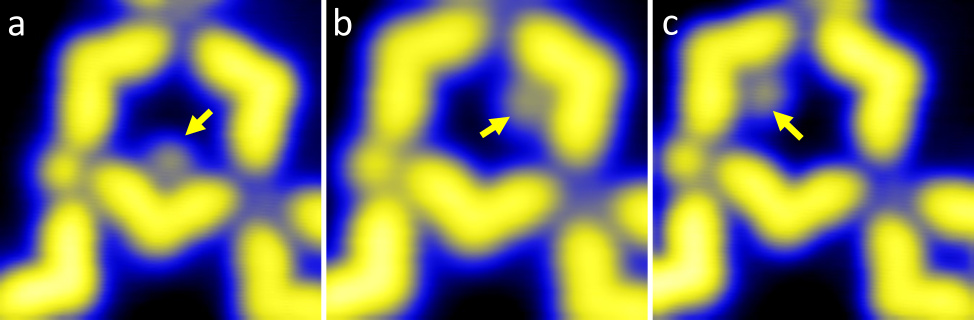
**Figure S9. (a-c)** After applying a voltage pulse at 2.0 V to the CO_2_ molecule marked by yellow arrows, it moves from one to another C3PC molecule in Fe-C3PC-ST on Au(111). Imaging parameters for all STM images: 3.4 nm × 3.4 nm; *V*_bias_ = 10 mV, *I* = 0.04 nA.


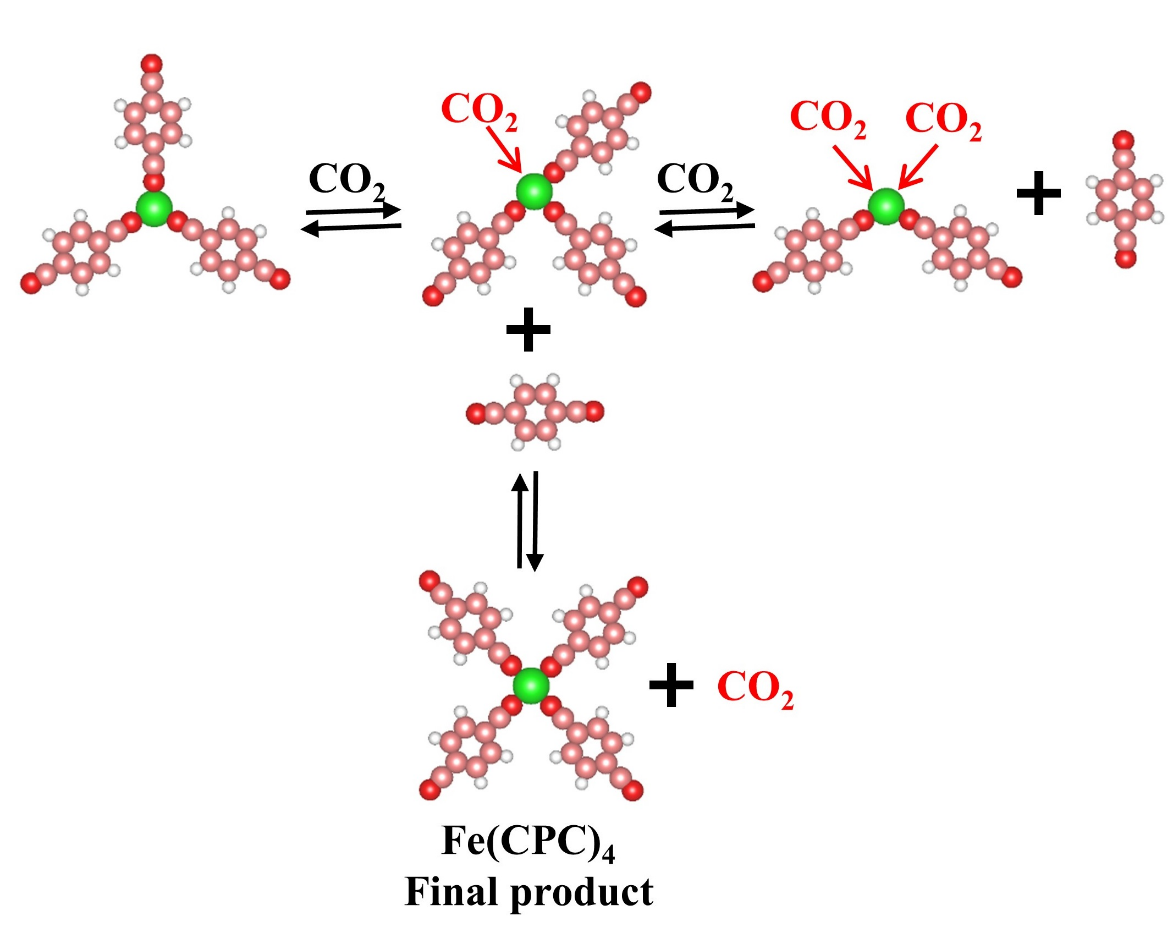
**Figure S10.** Scheme of structure transition from Fe(CPC)_3_ to Fe(CPC)_4_, which is used to understand the transformation from STs to chains via the CO_2_ catassembly process. According to Le Chatelier′s Principle, the final product is favorable when increasing the amount of CO_2_.


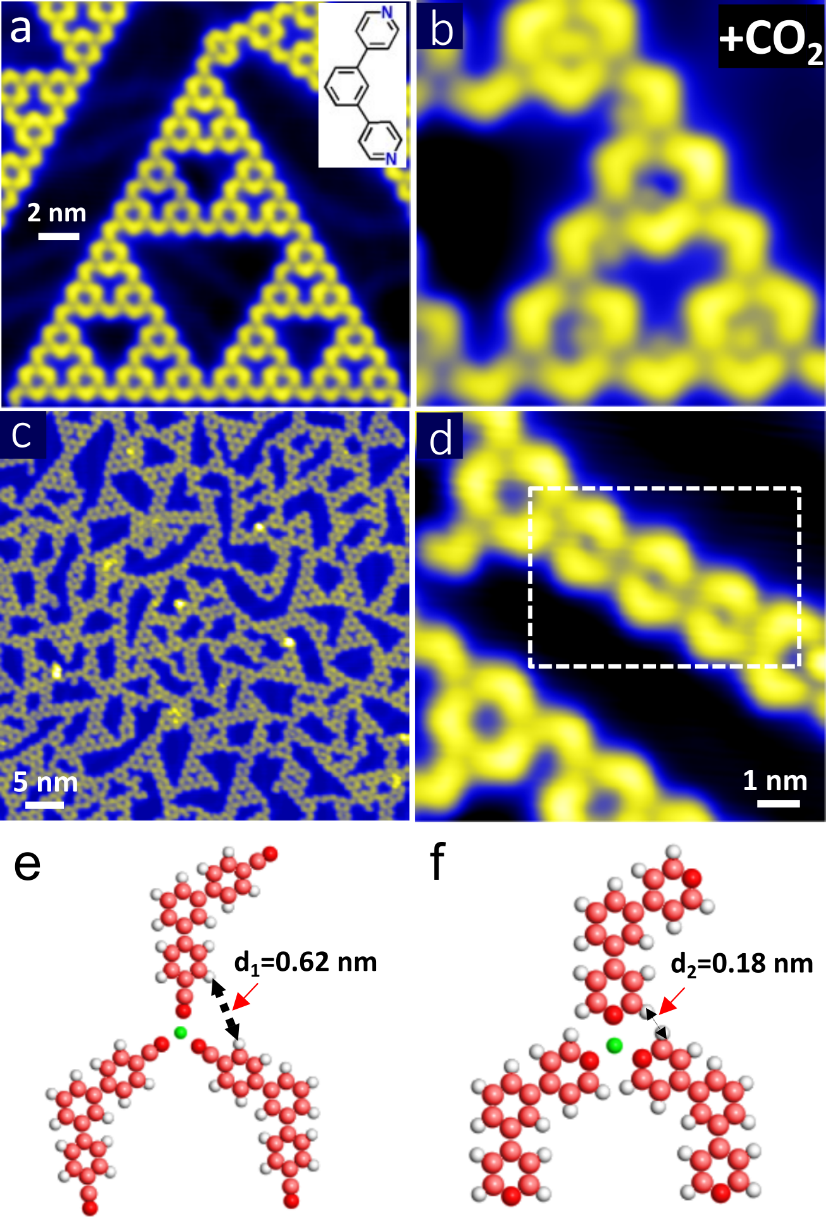
**Figure S11.** CO_2_ exposure on Fe-BPyB-STs. (**a**) Fourth-order Fe-BPyB-ST on Au(111) (20 nm × 20 nm). The chemical structure of BPyB is displayed in the upper right. (**b**) Second-order Fe-BPyB-ST after dosing CO_2_ of 60 L at around 30 K (6 nm × 6 nm). (**c**) STs after dosing CO_2_ of 650 L at room temperature (54 nm × 54 nm). (**d**) Enlarged STM image of Fe(BPyB)_4_ structures highlighted by the white dashed rectangle (7.6 nm × 7.6 nm). (**e** and **f**) Optimized molecular models of Fe(BPyB)_3_ and Fe(C3PC)_3_ nodes. The marked distances between two hydrogen atoms of neighbour molecules are 0.18 nm for Fe(BPyB)_3_ and 0.62 nm for Fe(C3PC)_3_, respectively. Imaging parameters: *I* = 0.04 nA, (a, b, d) *V*_bias_= 20 mV, (c) *V*_bias_ = 100 mV**.**
